# Alterations in Skeletal Muscle Insulin Signaling DNA Methylation: A Pilot Randomized Controlled Trial of Olanzapine in Healthy Volunteers

**DOI:** 10.3390/biomedicines12051057

**Published:** 2024-05-10

**Authors:** Kyle J. Burghardt, Paul R. Burghardt, Bradley H. Howlett, Sabrina E. Dass, Brent Zahn, Ahmad A. Imam, Abdullah Mallisho, Zaher Msallaty, Berhane Seyoum, Zhengping Yi

**Affiliations:** 1 Department of Pharmacy Practice, Eugene Applebaum College of Pharmacy and Health Sciences, Wayne State University, Detroit, MI 48201, USA; gl7527@wayne.edu (B.H.H.); fi2094@wayne.edu (S.E.D.); 2 Department of Nutrition and Food Science, Wayne State University, Detroit, MI 48202, USA; paul.burghardt@wayne.edu; 3 Department of Clinical Pharmacy, College of Pharmacy, University of Michigan, Ann Arbor, MI 48109, USA; zahnb@med.umich.edu; 4 Internal Medicine Department, College of Medicine, Umm Al-Qura University, Makkah 24381, Saudi Arabia; gm0670@wayne.edu; 5 Division of Endocrinology, School of Medicine, Wayne State University, Detroit, MI 48202, USA; ee5984@wayne.edu (A.M.); zmsallaty@wayne.edu (Z.M.); bseyoum@med.wayne.edu (B.S.); 6 Department of Pharmaceutical Science, Eugene Applebaum College of Pharmacy and Health Sciences, Wayne State University, Detroit, MI 48202, USA; zhengping.yi@wayne.edu

**Keywords:** epigenetic, antipsychotic, muscle

## Abstract

Antipsychotics are associated with severe metabolic side effects including insulin resistance; however, the mechanisms underlying this side effect are not fully understood. The skeletal muscle plays a critical role in insulin-stimulated glucose uptake, and changes in skeletal muscle DNA methylation by antipsychotics may play a role in the development of insulin resistance. A double-blind, placebo-controlled trial of olanzapine was performed in healthy volunteers. Twelve healthy volunteers were randomized to receive 10 mg/day of olanzapine for 7 days. Participants underwent skeletal muscle biopsies to analyze DNA methylation changes using a candidate gene approach for the insulin signaling pathway. Ninety-seven methylation sites were statistically significant (false discovery rate < 0.05 and beta difference between the groups of ≥10%). Fifty-five sites had increased methylation in the skeletal muscle of olanzapine-treated participants while 42 were decreased. The largest methylation change occurred at a site in the Peroxisome Proliferator-Activated Receptor Gamma Coactivator 1-Alpha (*PPARGC1A*) gene, which had 52% lower methylation in the olanzapine group. Antipsychotic treatment in healthy volunteers causes significant changes in skeletal muscle DNA methylation in the insulin signaling pathway. Future work will need to expand on these findings with expression analyses.

## 1. Introduction

Antipsychotics consist of a few classes of medications, including first-generation or typical antipsychotics, which are distinguished by their binding to the dopamine receptor. In addition, second-generation or atypical antipsychotics are characterized by a greater affinity for the serotonin receptor family. Antipsychotics are primarily used in the treatment of schizophrenia and bipolar disorder; however, their use has expanded to other areas as well [1,2,3]. Each class and agent presents with side effects including sedation, sexual dysfunction, weight gain and metabolic changes such as an increased risk of diabetes [4]. Some agents have more intensive metabolic side effects, such as olanzapine and clozapine, but nearly all antipsychotics cause a certain amount of weight gain and metabolic side effects [5,6,7,8,9].

Diabetes is a metabolic disease that, when not regulated, can lead to cardiovascular disease and a shortened life expectancy [10]. Insulin resistance, an early feature of diabetes, can occur both with and without obesity [11]. A primary tissue where insulin resistance develops is within the skeletal muscle [12,13]. The etiology of insulin resistance is multifactorial and can include defects in the insulin signaling pathway preventing proper insulin-stimulated glucose uptake through glucose transporter 4 (GLUT-4) [14]. Additionally, genetic mutations, for example in the peroxisome proliferator-activated receptor gamma (*PPARγ*) gene, may play a role in the development of insulin resistance by leading to altered insulin levels, molecular signaling and lipid metabolism. Finally, alterations in fatty acid metabolism and lipid accumulation within insulin-sensitive tissues are thought to contribute to insulin resistance as well [15]. In-depth reviews of insulin resistance are available [14,16,17,18]. Importantly, as the basis of this study, insulin resistance has been linked to antipsychotic treatment independent of mental illness, which itself has been correlated to glucose dysregulation [19,20,21,22].

The important connection between antipsychotics and metabolic side effects was brought to the forefront by the findings from the Clinical Antipsychotic Trials of Intervention Effectiveness (CATIE) studies and other studies which identified increased rates of metabolic syndrome, diabetes and insulin resistance in patients treated with antipsychotics [23,24,25]. This work has been continuously built upon with meta-analytic findings as well as deeper metabolic phenotyping, allowing for an in-depth investigation into the effect of antipsychotics on glucose regulation and insulin signaling [5,26,27,28]. Although the link between antipsychotic treatment and metabolic adverse effects was identified long ago, the cause of the connection remains unknown. One possible hypothesis could be that molecular changes, like that of DNA regulation, in the skeletal muscle lead to disruptions in the insulin signaling pathway and skeletal muscle glucose uptake. The potential mechanisms of antipsychotic-induced metabolic side effects are extensively reviewed elsewhere [29,30].

Pharmacoepigenetics has emerged as a scientific field to explain how the environment, in this case medication treatment, can influence gene regulation [31]. Epigenetic changes can be both heritable and transient, depending on the exposure, the gene and the location of the epigenetic change [32]. The main area of pharmacoepigenetic investigation is in DNA methylation (5-methylcytosine). In general, DNA methylation is correlated with gene repression while de-methylation is associated with increased gene expression [33]. DNA methylation provides additional means for genetic control by allowing different cell types, with the same underlying genetic code, to have varying gene expression patterns [34]. Importantly, for the study here, DNA methylation has been shown to be dysregulated in several disorders, including those of insulin resistance and diabetes, and dynamically influenced by medication treatment like antipsychotics [35,36].

The objective of this study was to investigate the possible connection between antipsychotic medication treatment and changes in skeletal muscle DNA methylation of the insulin signaling pathway in healthy volunteers using a randomized controlled trial of olanzapine. Olanzapine was chosen since it is the antipsychotic, along with clozapine, with the greatest metabolic risk and therefore a candidate agent for studies on the effect of antipsychotics on insulin-sensitive tissues. Furthermore, olanzapine has an increased safety profile compared to clozapine because it does not carry the same risks of hematologic side effects. Healthy volunteers were recruited to investigate the direct effects of antipsychotic medication on skeletal muscle DNA methylation independent of the possible effects of mental illness associated with glucose dysregulation discussed above. We hypothesized that treatment of healthy volunteers with olanzapine for 7 days would result in a dysregulated skeletal muscle DNA methylation profile within the insulin signaling pathway compared to placebo-treated volunteers. The long-term goal of this study is to identify mechanisms and biomarkers of antipsychotic-induced insulin resistance that will allow better treatment protocols and individualized medicine aimed at maximizing medication outcomes.

## 2. Materials and Methods

### 2.1. Participant Population

Participants were recruited from the metropolitan Detroit area via approved flyers and internet postings from February to August 2017. Participants were physically and mentally healthy with the following inclusion criteria: (1) 21–45 years old, (2) body mass index between 18.5 and 24.9 kg/m^2^, (3) no history of drug or alcohol dependence or abuse, (4) no current or past history of psychiatric or neurologic disease, (5) minimal exercise routine prior or during study (light walking allowed), (6) normal liver function tests, and (7) minimal alcohol intake (<1 drink per day). Potential participants were excluded if they: (1) had a 1^st^-degree relative with diabetes, (2) had current or recent nicotine intake, (3) presence of organic/physical disease, (4) currently taking prescription or over-the-counter medications that could affect glucose, (5) currently pregnant or lactating, (6) history of unstable weight, and (7) personal or family history of cardiac arrhythmias or seizures (patients were not required to undergo an electrocardiogram). All participants gave written, informed consent to participate in the study, which was approved by the Wayne State University Institutional Review Board and registered on clincaltrials.gov (NCT02708394).

### 2.2. Study Procedures and Assessments

Participants were initially invited to the Wayne State University Clinical Research Center to undergo informed consent followed by a screening visit that included a survey form to assess demographic factors and medical history as well as a psychiatric interview via the Mini-International Neuropsychiatric Interview (MINI) to rule out current or historical physical and psychiatric illness [37]. During the screening session, body mass index was measured and a blood draw was performed to assess liver function as a safety measure due to the study drug’s metabolism by the liver.

After the screening visit, if the participant met inclusion/exclusion criteria, they were invited back to the clinical research center to be enrolled in the study, randomized in parallel form to drug or placebo treatment, and complete two final visits (a treatment baseline visit and follow-up visit). At the treatment baseline visit, participants were assigned to olanzapine or placebo treatment for 7 days using a double-blind, randomized 1:1 procedure in blocks of 4. The olanzapine dosing schedule was as follows: nights 1 and 2, 5 mg followed by 10 mg for nights 3–7. Allocation and drug blinding were performed by study personnel responsible for preparing the study drug but otherwise not involved in any other aspect of the study. Also, during this baseline visit, participants underwent indirect calorimetry, an intravenous glucose tolerance test (IVGTT) and anthropometric testing. The IVGTT proceeded with a 0.3 g/kg glucose push at time 0 followed by a 0.02 U/kg push of insulin at time 20. Blood sampling was taken every 1 to 10 min for 3 h to model insulin sensitivity using the minimal model (MINMOD) technique with MINMOD Millennium software 6.03 [38,39]. Glucose was measured by a bedside YSI 2300 Stat Plus glucose analyzer (YSI, Yellow Springs, Ohio, USA), and insulin was assayed using the ALPCO Insulin Enzyme-linked Immunosorbent Assay (ELISA) assay kit on a Perkin Elmer EnSight multimode microplate reader (PerkinElmer, Shelton, Connecticut, USA) in duplicate. Insulin values were converted from uIU/L to pmol/L by multiplying by 6.00 pmol/L [40]. The parameters derived from the MINMOD IVGTT include the insulin sensitivity index (SI), disposition index (DI, a measure of islet cell ability to secrete insulin correcting for insulin sensitivity), the acute insulin response to glucose (AIRg), and glucose effectiveness (Sg). The calculation of SI is comparable to the gold-standard values of the hyperinsulinemic euglycemic clamp [41,42]. Additionally, the homeostatic assessment model (HOMA) and beta cell function were calculated according to Matthews and colleagues by the MINMOD software 6.03 [43]. Indirect calorimetry utilized a Parvo Medics TrueOne 2400 system (Parvo Medics, Salt Lake City, UT, USA) to calculate resting metabolic rate with the participant lying supine in a dark, quiet room for 30 min [44]. On the morning of day 8 (follow-up visit), the participants came in to undergo a muscle biopsy using the modified Bergstrom technique to be used for DNA methylation analysis [45]. The modified Bergstrom technique utilizes a specialized biopsy needle under local anesthesia by a certified, trained medical physician to obtain 20–50 mg of skeletal muscle tissue from the vastus lateralis. Volunteers lay in a recumbent position at rest for 5 min prior to the procedure. A certified nurse and physician prepped the biopsy site, administered local anesthesia, and performed the biopsy, which lasted approximately 5 min. Biopsied muscle was immediately cleaned of blood with phosphate-buffered saline and flash-frozen in liquid nitrogen until further processing. Possible adverse effects of biopsies include pain or bruising at the biopsy site, bleeding at the biopsy site and infection. Participants also repeated the indirect calorimetry and anthropometric measurements. Participants came in for all visits in a fasting state (>8 h). Steps were measured with an Actigraph wGT3X-BT (ActiGraph, Pensacola, FL, USA), and hunger was measured by a 100 mm visual analog scale [46,47].

Adverse effects from study treatment were assessed using the Glasgow Antipsychotic Side-Effect Scale (GASS) at days 2, 4, 6 and 7 [48]. The GASS assesses a series of antipsychotic-related side effects (22 total) and grades them on frequency. Each GASS was summed to provide a global side-effects rating from absent/mild to severe. Questions 1–20 receive points ranging from 0 (never) to 3 (experiencing a side effect every day), and questions 21 and 22 can receive 3 points (yes, experienced) or 0 points (no, did not experience).

### 2.3. DNA Methylation Analysis

For the primary outcome of DNA methylation changes in the insulin signaling pathway, approximately 10–15 mg of the frozen, biopsied muscle was pulverized using a bead homogenizer, and genomic DNA was extracted with the Qiagen AllPrep DNA Mini Kit (Qiagen, Hilden, Germany) on an automated Qiagen Qiacube machine (Qiagen, Hilden, Germany). Extracted DNA quantity was measured on a Qubit Fluorometer (Thermo Fisher Scientific, Waltham, MA, USA), and 500 ng were bisulfite-converted using the Qiagen Epitect kit (Qiagen Hilden, Germany). The bisulfite-converted DNA was then utilized for DNA methylation analysis with the Illumina HumanMethylation EPIC array (Illumina, San Diego, CA, USA) through the Wayne State University Applied Genomic Technology Core. Raw IDAT files from the EPIC array were returned and analyzed using the CHAMP package in R statistical software 4.2.1 [49]. In brief, after loading the IDAT files, data were pre-processed to remove: (1) probes with a detection *p*-value less than 0.01, (2) probes with less than 3 beads in at least 5% of samples per probe, (3) non-CpG probes, (4) probes close to single nucleotide polymorphisms, (5) probes that may tag multiple genomic locations and (6) probes on sex chromosomes [50,51]. These filtered data were then normalized using the beta-mixture quantile approach and batch-corrected using the ComBat package [52,53]. The singular value decomposition method was employed to identify the number and extent of significant components in the dataset to assist with identifying non-biological sources of variation such as batch effects and the effect of batch correction during normalization [54]. These steps yielded the pre-processed, normalized and batch-corrected epigenomic dataset for further analysis.

Within this study, we sought to focus our epigenomic analyses on the candidate pathway of insulin signaling, as our primary objective was to investigate the effects on olanzapine treatment on human skeletal muscle insulin signaling to begin connecting skeletal muscle molecular changes to atypical-antipsychotic-induced insulin resistance. To achieve this, we first extracted all genes listed for the Kegg pathway of “insulin signaling” (map04910). This pathway contains a total of 137 genes. We then obtained all probes from the EPIC array manifest associated with each of these 137 genes. This yielded a total of 6470 probes in the insulin signaling pathway for analyses using the pre-processed and normalized dataset described above. To that end, we applied a filtering step to only include the identified 6470 probes in our analyses by removing all other probes from the pre-processed and normalized dataset.

### 2.4. Statistics

For this pilot/feasibility study, a sample size calculation estimated that 6 participants per group achieved 87% power assuming an effect size of 2.0 and an alpha of 0.05 for DNA methylation. Such an effect size would allow for a 10% difference in methylation assuming a standard deviation of 5%. For the demographic and clinical variables, one-way analysis of variance (ANOVA) was utilized for normally distributed variables, and chi-square tests were utilized for categorical variables, as appropriate in JMP 17 software. Normality of variables was tested using the Shapiro–Wilk Goodness of Fit test. Differences in clinical variables were analyzed using the matched pairs model in JMP, which incorporates baseline levels in the analysis as a control with a non-parametric Wilxocon Signed-Rank Test for non-normal variables. For the primary analysis of DNA methylation, insulin signaling probes were considered significant between participants treated with olanzapine and placebo if they achieved a false discovery rate (FDR)-corrected *p*-value less than 0.05 and a beta value (measure of % methylation) difference between the groups of ≥10%. These cutoffs were used to control the family-wise error rate while also providing probes that have meaningful differences in DNA methylation.

## 3. Results

### 3.1. Study Flow, Patient Characteristics and Clinical Measurements

A total of 14 participants consented to participate. One participant withdrew for unknown reasons, prior to being randomized for drug treatment, and one participant withdrew due to sedation. The latter participant was assigned to olanzapine. Twelve participants (six placebo, six olanzapine) completed both visits and all procedures, had an average age of 25.42 ± 4.17, and 41.67% were female. The average age and sex in the olanzapine group was 23.83 ± 0.98 and 33.33% female, while the average age and sex was 27.00 ± 5.59 and 50% female in the placebo group. There were no significant differences in demographics or baseline variables between the groups at baseline (Table 1).

Following seven days of treatment with olanzapine or placebo, the groups significantly differed for SI (the insulin sensitivity index, *p* = 0.034). Although not statistically significant, trends (*p* ≤ 0.10) were observed for weight, BMI, fasting glucose, DI, resting energy expenditure and average steps per day. Details of the comparisons of clinical variables are in Table 2.

### 3.2. Adverse Effects

Eleven of the twelve participants, regardless of assigned treatment, rated side effects as absent or mild. One participant, based on the GASS scoring, rated their side effects as “moderate”. This participant was in the placebo group. Of note, the single participant who withdrew due to sedation described above rated their side effect as mild. There were no within-group differences observed from day 2 to day 7. An overview of adverse effects is provided in Table 3. There were no significant differences between the groups at any of the time points. Of note, there were no significant adverse effects from the biopsy procedure during the study.

### 3.3. Methylation Analyses

Skeletal muscle DNA methylation within the insulin signaling pathway was analyzed after receiving either olanzapine or placebo treatment for 7 days. The quality figures are presented in the Supplementary Information (Supplementary Figures S1–S4) which indicate the pre and post effects of the normalization strategy as well as the success of the batch correction. After pre-processing and normalization, a total of 5483 methylation sites (84.74% of array manifest sites) were available for analysis in the insulin signaling pathway. A total of 97 methylation sites were significant based on an FDR cutoff of 0.05 and beta value difference of at least 10%. The greatest beta-value difference was 24.9% for hypermethylated (i.e., genes whose methylation increased in the olanzapine group) genes and 52.1% for hypomethylated genes. Fifty-five sites had increased methylation in the skeletal muscle of olanzapine-treated participants while 42 had decreased. The top 30, based on beta value, are presented in Table 4, and the full results are presented in Supplementary Table S1. In terms of enrichment of genes found in the 97 significant sites, 33 genes are only represented once, while 18 genes are represented by multiple sites. The gene with the highest proportion of significant sites, based on the number of sites available for analysis per gene, was the Ribosomal Protein S6 Kinase B1 (*RPS6KB1),* which had 16.7% (one significant site of six total sites analyzed). The top 20 enriched genes are presented in Table 5. Of these top 20 genes, Mitogen-Activated Protein Kinase 8 (*MAPK8)*, Insulin Receptor Substrate 1 (*IRS1)* and MAPK Interacting Serine/Threonine Kinase 1 (*MKNK1)* had multiple sites all showing the same direction of methylation change (*MAPK8* and *MKNK1* all increased and *IRS1* all decreased). Of note, although the Regulatory Associated Protein of MTOR Complex 1 (*RPTOR*) gene had nine sites all with hypermethylation in the olanzapine group, the *RPTOR* had a total of 476 sites in the analysis, meaning that 8.7% of *RPTOR*’s sites met statistical significance. The complete table detailing the enrichment of all genes found among the 97 significant sites is in the Supplementary Table S2. Additional figures depicting the overall landscape of methylation for the insulin signaling pathway as well as for several of the top significant genes with multiple significant sites can be found in the Supplementary Materials.

## 4. Discussion

This analysis of the skeletal muscle insulin signaling epigenome in healthy volunteers treated with olanzapine or placebo for 7 days indicates that olanzapine causes significant alterations in 5-methylcytosine DNA methylation. Furthermore, in participants treated with olanzapine, their insulin sensitivity index estimated from the IVGTT decreased significantly. The methylation results found a total of 97 methylation sites from 51 genes that were statistically significant and showed at least a 10% methylation difference between the olanzapine and placebo groups. Approximately 55% showed increased methylation in the olanzapine group, and the gene with the greatest absolute difference was the Peroxisome proliferator-activated receptor Gamma Coactivator 1-Alpha (*PPARGC1A*) gene, which had, on average, a 52% lower methylation in the olanzapine group. The methylation analyses also found several genes with uniform hypo or hypermethylation across all significant sites in the olanzapine group relative to the placebo group.

The *PPARGC1A* gene, which encodes for a transcription factor called PGC-1α, interacts with the PPARgamma receptor leading to multiple interactions and pathway effects on energy metabolism, gluconeogenesis and mitochondrial biogenesis [55,56]. Within the skeletal muscle, there is evidence that *PPARGC1A* is involved in a wide range of pathways and cellular activities including glucose metabolism, inflammation, lipid metabolism, mitochondrial gene regulation, thermogenesis, and myokine secretion [57,58]. In addition to being suggested as a general drug target for neuropsychiatric disorders, metabolic-based investigations into the effects of antipsychotics on *PPARGC1A* have found some important associations [59]. Sarsenbayeva and colleagues investigated the effect of olanzapine and aripiprazole on isolated and cultured subcutaneous adipocytes from healthy volunteers [60]. Within their study, 72 h of elevated concentrations of olanzapine and risperidone, and therapeutic concentrations of olanzapine, reduced mRNA expression of *PPARGC1A* by 0.66, 0.71 and 0.80 (fold-change values), respectively. Similar reductions in the protein encoded by *PPARGC1A*, PGC-1α, were found in the brown fat of mice treated with risperidone or olanzapine versus controls and the brown fat of rats treated with olanzapine [61,62,63]. Within the study here, methylation of *PPARGC1A* was decreased by 52% in the skeletal muscle of the olanzapine group which, given the general known correlation of decreased methylation with increased gene expression, suggests opposite effects from those described in the adipose tissue above. Interestingly, studies have found paradoxical effects of increased muscle PGC-1α protein expression (the product of *PPARGC1A*) whereby increased PGC-1α was correlated with mitochondrial biogenesis but reduced insulin-stimulated glucose uptake [64]. The authors posit that this may be due to intracellular lipid accumulation, which has been shown to occur with antipsychotics in insulin-sensitive tissues [21]. To our knowledge, our finding is the first to demonstrate the effect of antipsychotic treatment on metabolic tissue methylation of *PPARGC1A* and the first in the skeletal muscle. Future studies will need to assess skeletal muscle mRNA expression and its correlation to the observed methylation changes identified here as well as to perform simultaneous evaluations of methylation changes in skeletal muscle and adipose tissue to understand how these two important peripheral insulin-sensitive tissues are influenced by antipsychotic treatment.

The second-most changed methylation site, based on the difference of beta between the groups, was Protein Kinase CAMP-Dependent Type I Regulatory Subunit Beta (*PRKAR1BI*), which codes for a regulatory subunit of protein kinase A involved in cyclic AMP activity. One of the primary downstream interactions of dopamine receptors is with protein kinase A; thus, there is a theoretical overlap between this gene and the activity of antipsychotics at the dopamine receptor [65]. The *PRKAR1BI* showed 25% increased methylation in the antipsychotic group within the study here, suggesting a potential downregulation of the gene. Studies have shown correlations between peripheral dopamine activity and skeletal muscle insulin sensitivity, and therefore it may be hypothesized that this gene’s decreased activity interacts with peripheral dopamine pathways to influence skeletal muscle physiology and insulin signaling [66].

The final gene with a greater than 20% change between the olanzapine and placebo group was the *RPTOR* gene, which had a 21% increase in the olanzapine group. Involved in the regulation of cell growth secondary to insulin and other nutrient signaling due to its close association with the mammalian target of rapamycin (mTOR), *RPTOR* has been implicated in the side effects of antipsychotics, including weight gain and extrapyramidal symptoms [67,68,69]. A possible effect of increased methylation of the *RPTOR* gene could be decreased gene and protein expression leading to aberrant skeletal muscle physiology and metabolic regulation, including reduced oxidative capacity, increased dystrophy and higher glycogen stores [70,71].

There have been a few studies assessing the skeletal muscle insulin signaling pathway in the context of antipsychotic treatment whose findings can be compared to the findings presented here. In a study by Engl and colleagues, treatment of L6 skeletal muscle cells with olanzapine inhibited insulin receptor substrate 1 (IRS-1)-associated phosphoinositide 3-kinase (PI3K) activity by reducing the activity of IRS-1, protein kinase (AKT) and glycogen synthase kinase-3 (GSK-3) through reduced tyrosine phosphorylation [72]. However, they did not identify altered individual protein abundance. Similar reductions in AKT phosphorylation were identified in C2C12 myotubes with clozapine, C2C12 myotubes with olanzapine and in L6 cells with clozapine [73,74,75]. However, different models have identified differing effects. For example, in a study by Castellani and colleagues using glucagon receptor-deficient mice, it was found that AKT phosphorylation was not affected by olanzapine administration, and therefore, altered phosphorylation was not able to explain the protective effect of this knockout model against olanzapine-induced glucose dysregulation [76]. Furthermore, in a study of quetiapine, an antipsychotic with reduced metabolic liability as compared to olanzapine, it was found that there was no effect of quetiapine treatment on mouse muscle *INSR*, *IRS1*, *Pik3r1* or *AKT2* mRNA levels, although a trend was observed for *Pik3r1* (decrease) and *AKT2* (increase) [77]. Additionally, in a study of the beneficial effects of exercise on antipsychotic-induced glucose dysregulation, Boyda and colleagues identified no effect of olanzapine on exercise-induced GLUT4 protein muscle abundance in rats, suggesting that olanzapine administration does not inhibit the positive effects of exercise on glucose tolerance [78]. Finally, in the only other human study to date, investigators identified an increase in skeletal muscle methylation for *AKT1* and *AKT2* in bipolar patients on atypical antipsychotics compared to patients on mood stabilizers [79]. In comparison to the changes in insulin signaling described above, we report here that of the 97 significant genes, *AKT2* was represented with one significant site showing increased methylation in the olanzapine group, and *PI3KR* was represented with two significant sites, both showing a decrease in methylation in the olanzapine group. These changes are suggestive of a decrease in AKT2 levels and an increase in PI3K levels, which does mirror some of the findings described above; however, future studies would need to confirm such interpretations with objective measurements of mRNA and protein levels as well as protein activity.

Overall, the findings detailed in the study presented in this paper have some similarities but many differences to the literature investigating insulin signaling pathway changes in insulin-sensitive tissues secondary to antipsychotic treatment described in the preceding paragraph. One important aspect to consider when comparing the results of the study here with other findings is the study design being utilized. The study here used a shorter administration (7 days) of olanzapine in healthy volunteers to understand the acute effects of antipsychotics on skeletal muscle DNA methylation. Others have utilized animal models, differing antipsychotics (e.g., clozapine, aripiprazole, etc.) and differing treatment lengths and dosing strategies. The study here is an important step in beginning to translate previous pre-clinical findings to humans, but future investigations will need to understand acute versus chronic effects of antipsychotic treatment on insulin-sensitive tissues to ultimately identify mechanisms that can be targeted to reduce the potential metabolic liability of antipsychotics.

This study was strengthened by its randomized, controlled design and use of healthy volunteers to study causal epigenetic mechanisms in humans; however, a few limitations should be considered. First, the trial had a small number of participants owing to the intense nature of the phenotyping involved and the muscle biopsy procedure to acquire tissue for epigenetic analysis. This small sample size likely contributed to the lack of significant differences in the clinically measured variables. Only the variable of SI was statistically significant (*p* = 0.034) from the IVGTT while DI was a possible trend (*p* = 0.11). The reasons for this beyond a limited sample size could be that a 7-day antipsychotic administration influences specific MINMOD-calculated parameters of the IVGTT such as insulin sensitivity (SI) but not the acute response to insulin (AIRg). In the only other study of antipsychotic effects on IVGTT parameters in healthy volunteers to date, Hahn and colleagues found a single dose of olanzapine only significantly affected Sg (glucose effectiveness) but no other parameter of the IVGTT. Further studies are needed to investigate the time-dependent effects of antipsychotic administration on IVGTT parameters. Nevertheless, we were able to identify 97 significant genes using conservative cutoff criteria consisting of multiple testing correction and methylation difference between the groups. These findings still require further validation using a measurement of functional gene changes such as gene expression or protein abundance and activity. Furthermore, the limited number of subjects reduced the power to perform correlational analyses between DNA methylation and metabolic measurements. The measurement of insulin sensitivity used in this study, the IVGTT, is unable to capture some aspects of glucose tolerance that may be captured with more intense measures like the hyperinsulinemic euglycemic clamp. The IVGTT does show good correlation to the clamp as a measurement of insulin sensitivity while also providing additional measurements of glucose tolerance such as acute insulin response. Future studies could provide additional information by assessing gene changes during insulin stimulation such as with the clamp. Within this study, we chose to focus our epigenetic analyses on a candidate pathway. It is possible that antipsychotic effects that are associated with insulin resistance could reach beyond the insulin signaling pathway due to the unique aspects of these drugs. Future studies should consider other skeletal muscle function pathways as well as novel pathways to fully understand how antipsychotics cause insulin resistance and other metabolic side effects.

## 5. Conclusions

In conclusion, this study identified a series of methylation changes within the insulin signaling pathway that occur in the skeletal muscle in healthy volunteers treated with olanzapine compared to placebo. These findings could lead to further mechanistic insight, biomarker development or the creation of interventions that target this pathway and reverse or prevent the methylation changes observed here. The findings here represent another step in understanding how antipsychotics cause insulin resistance to prevent medication-induced comorbidity while preserving the therapeutic effects of antipsychotics.

## Figures and Tables

**Table 1:** Baseline Demographics and Variables.

Baseline Variable	Olanzapine Baseline	Placebo Baseline	Between-Group *p*-Value
Age	23.83 ± 1.00	27.00 ± 5.59	0.20
Sex (% female)	33	50	0.56
Weight	64.07 ± 8.63	64.87 ± 4.75	0.85
BMI	21.79 ± 2.28	21.57 ± 1.32	0.85
Fasting Glucose	82.65 ± 4.67	86.62 ± 4.91	0.18
Fasting Insulin	35.09 ± 16.06	26.41 ± 9.97	0.29
HOMA-IR	1.18 ± 0.49	0.95 ± 0.38	0.39
Beta Cell	151.95 ± 157.79	75.80 ± 34.00	0.32
AIRg	740.83 ± 472.21	542.12 ± 410.72	0.48
DI ^&^	3260.07 ± 1511.27	1855.06 ± 1326.76	0.14
Sg	0.034 ± 0.014	0.021 ± 0.0094	0.11
SI	6.18 ± 3.65	3.82 ± 2.47	0.49
Resting Energy Expenditure ^&^	1361.37 ± 239.81	1368.33 ± 106.03	0.95
Hunger Score Day 2	6.96 ± 0.43	6.68 ± 1.38	0.64

^&^ Variable was not normal with Shapiro–Wilk Goodness of Fit Test; therefore, non-parametric test utilized.

**Table 2:** Post-Treatment Variables.

Endpoint Variable	Olanzapine Endpoint	Placebo Endpoint	Between-Group *p*-Value
Weight (kg)	64.83 ± 8.53 ^a^	65.00 ± 4.98	0.052
BMI	22.06 ± 2.33 ^a^	21.62 ± 1.42	0.060
Fasting Glucose	86.00 ± 7.59	85.62 ± 4.65	0.089
Fasting Insulin	8.06 ± 4.06	7.30 ± 2.26 ^a^	0.57
HOMA-IR	1.68 ± 0.76	1.55 ± 0.51 ^a^	0.69
Beta Cell	175.05 ± 187.90	130.47 ± 32.76	0.11
AIRg	953.50 ± 682.95	692.67 ± 533.82	0.93
DI ^&^	2325.54 ± 1182.02	2654.15 ± 1257.78	0.10
Sg	0.028 ± 0.010	0.022 ± 0.013	0.34
SI	3.55 ± 2.26	6.14 ± 5.36	0.034 *
Resting Energy Expenditure ^&^	1519.20 ± 486.10	1194.40 ± 107.60	0.078
Hunger Score Day 6	7.69 ± 1.32	6.16 ± 0.61	0.13
Average Steps Per Day	3884.72 ± 1702.32	2322.08 ± 1197.18	0.096

^a^ Significant change within-group difference (*p*<0.05); ^&^ variable was not normal with Shapiro–Wilk Goodness of Fit Test; therefore, non-parametric test utilized. * indicated statistically significant *p* < 0.05.

**Table 3:** Side-Effects Data for Treatment Groups.

Adverse Effect Variable	Day 2	Day 4	Day 6	Day 7
Olanzapine GASS Score	8.83 ± 5.12	10.33 ± 5.01	9.00 ± 3.58	9.33 ± 5.16
Placebo Gass Score	4.00 ± 1.73	5.67 ± 6.91	6.67 ± 9.29	6.33 ± 10.05
All Participant GASS Score	6.42 ± 5.14	8.00 ± 6.25	7.83 ± 6.82	7.83 ± 7.78
*p*-value for Group Comparison	0.10	0.21	0.58	0.53

**Table 4:** Top 30 Significant Skeletal Muscle Insulin Pathway Methylation Sites Between Olanzapine and Placebo Groups.

Gene	Methylation Site Number	Raw *p*-Value	FDR Corrected *p*-Value	Delta Beta *	Gene Feature	CG Type
*PPARGC1A*	cg24160354	4.05 × 10^−3^	1.58 × 10^−2^	−0.52	Body	Opensea
*PRKAR1B*	cg24368702	1.36 × 10^−5^	2.40 × 10^−4^	0.25	5’UTR	Shelf
*RPTOR*	cg09803959	4.50 × 10^−6^	1.09 × 10^−4^	0.21	Body	Shore
*HK1*	cg23177739	1.68 × 10^−6^	5.42 × 10^−5^	−0.20	Body	Opensea
*RAF1*	cg25055867	1.68 × 10^−3^	8.13 × 10^−3^	0.19	Body	Opensea
*PRKACA*	cg19586199	5.26 × 10^−4^	3.43 × 10^−3^	−0.19	TSS200	Shelf
*CBLB*	cg15276228	3.46 × 10^−3^	1.42 × 10^−2^	−0.18	Body	Opensea
*PRKCZ*	cg17156349	3.31 × 10^−4^	2.41 × 10^−5^	0.17	Body	Opensea
*PRKAG2*	cg21764708	1.86 × 10^−8^	2.50 × 10^−6^	0.17	5’UTR	Opensea
*MKNK1*	cg25263021	4.50 × 10^−6^	1.09 × 10^−4^	0.17	Body	Opensea
*BRAF*	cg10155158	2.05 × 10^−5^	3.15 × 10^−4^	0.16	Body	Opensea
*TSC2*	cg16424182	1.65 × 10^−5^	1.45 × 10^−3^	0.16	Body	Shore
*PRKAG2*	cg21008208	3.49 × 10^−7^	1.80 × 10^−5^	0.16	Body	Opensea
*RHEB*	cg07943849	4.41 × 10^−6^	1.08 × 10^−4^	0.16	Body	Opensea
*PIK3R1*	cg25091228	3.43 × 10^−5^	4.64 × 10^−4^	−0.16	TSS200	Shore
*SREBF1*	cg13891611	6.99 × 10^−9^	1.47 × 10^−6^	−0.16	Body	Shelf
*EIF4E1B*	cg07135540	4.22 × 10^−9^	1.20 × 10^−6^	0.15	TSS1500	Shore
*PTPRF*	cg15620905	3.32 × 10^−7^	1.77 × 10^−5^	0.15	Body	Opensea
*FOXO1*	cg07109046	3.31 × 10^−9^	1.20 × 10^−6^	0.15	Body	Opensea
*INPP5A*	cg11145302	0.004125	1.61 × 10^−2^	0.14	Body	Shore
*PRKAR2A*	cg01004980	2.50 × 10^−5^	3.69 × 10^−4^	−0.14	Body	Shore
*MAPK10*	cg05045427	9.90 × 10^−8^	7.75 × 10^−6^	−0.14	5’UTR	Opensea
*SREBF1*	cg17029706	9.87 × 10^−9^	1.87 × 10^−6^	−0.14	Body	Opensea
*IRS1*	cg22612792	1.15 × 10^−3^	6.01 × 10^−3^	−0.14	3’UTR	Opensea
*IRS1*	cg14283647	1.13 × 10^−2^	3.45 × 10^−2^	−0.14	1stExon	Shore
*PRKAG2*	cg01005180	1.30 × 10^−6^	4.58 × 10^−5^	0.14	Body	Opensea
*RPTOR*	cg05249744	3.67 × 10^−3^	1.48 × 10^−2^	0.14	Body	Shelf
*RPTOR*	cg22636722	3.05 × 10^−3^	1.29 × 10^−2^	0.14	Body	Shore
*RPTOR*	cg24667756	2.51 × 10^−5^	3.69 × 10^−4^	0.14	Body	Shelf
*PRKCI*	cg21140290	1.06 × 10^−5^	2.01 × 10^−4^	0.14	Body	Opensea

Table provides Gene name, CG site number, FDR-corrected *p*-value, delta beta value, gene feature and CG type provided. A total of 97 sites were found to be significant in this study based on an FDR < 0.05 and delta beta ≥ 10%. * A negative delta beta indicates a decrease in the olanzapine group. Complete list of significant sites is in Supplementary Table S1.

**Table 5:** Top 20 Enriched Genes for the 97 Significant Sites. The table provides the enrichment or proportion of significant sites for the 97 significant sites detailed in Table 3 along with the total available sites for analysis and the directionality of change in the olanzapine group relative to the placebo group. For the direction of change, a single arrow or “all” indicates the change for that single site, or all sites were in the same direction. A number followed by an arrow indicates the number of genes changed in that direction. For example, 1↑ 4↓ indicates that 1 site was increased in the olanzapine group and 4 were decreased in the olanzapine group relative to the placebo group.

Gene	Number of Significant Probes	Proportion of Total Probes Analyzed within Gene	Direction of Methylation Change
*RPS6KB1*	1	16.7	↑
*PPP1R3A*	1	14.3	↓
*MAPK8*	3	11.1	All ↑
*SREBF1*	5	10.9	1↑ 4↓
*IRS1*	4	10.8	All ↓
*PHKG1*	1	9.1	↓
*RHEB*	3	8.6	2↑ 1↓
*PYGM*	2	7.4	1↑ 1↓
*CALML6*	1	7.1	↓
*PRKAR2A*	1	7.1	↓
*MAPK10*	4	7.0	2↑ 2↓
*MKNK1*	2	6.3	All ↑
*PKLR*	1	6.3	↓
*HK1*	4	6.0	3↑ 1↓
*CALM3*	1	5.3	↑
*CBL*	1	5.0	↓
*GCK*	2	4.8	1↑ 1↓
*PTPN1*	2	4.5	1↑ 1↓
*CRK*	1	4.5	↓
*SLC2A4*	1	4.3	↑

## Data Availability

The original contributions presented in the study are included in the article/Supplementary Materials, further inquiries can be directed to the corresponding author/s.

## Supplementary Materials

The following supporting information can be downloaded at: https://www.mdpi.com/article/10.3390/biomedicines12051057/s1, Figure S1: Pre-Normalization Probe Type Density Plot; Figure S2: Post-Normalization Probe Type Density Plot; Figure S3: Pre-Batch Correction Singular Value Decomposition Plot; Figure S4: Post-Batch Correction Singular Value Decomposition Plot; Figure S5: CG Type Proportions for 97 Significant Sites; Figure S6: Gene Feature Proportions for 97 Significant Sites; Figure S7; Gene Methylation Figure for *PRKAR1B*; Figure S8: Gene Methylation Figure for *SREBF1*; Figure S9: Gene Methylation Figure for *IRS1*; Table S1: Full Methylation Results; Table S2: Enrichment Table Results.
