# Supplementary material for: Alterations in Skeletal Muscle Insulin Signaling DNA Methylation: A Pilot Randomized Controlled Trial of Olanzapine in Healthy Volunteers
Source: Biomedicines. 2024 May 10;12(5):1057. doi: 10.3390/biomedicines12051057 (PMC11117943; doi:10.3390/biomedicines12051057)

Figure S1 – Pre-Normalization Probe Type Density Plot

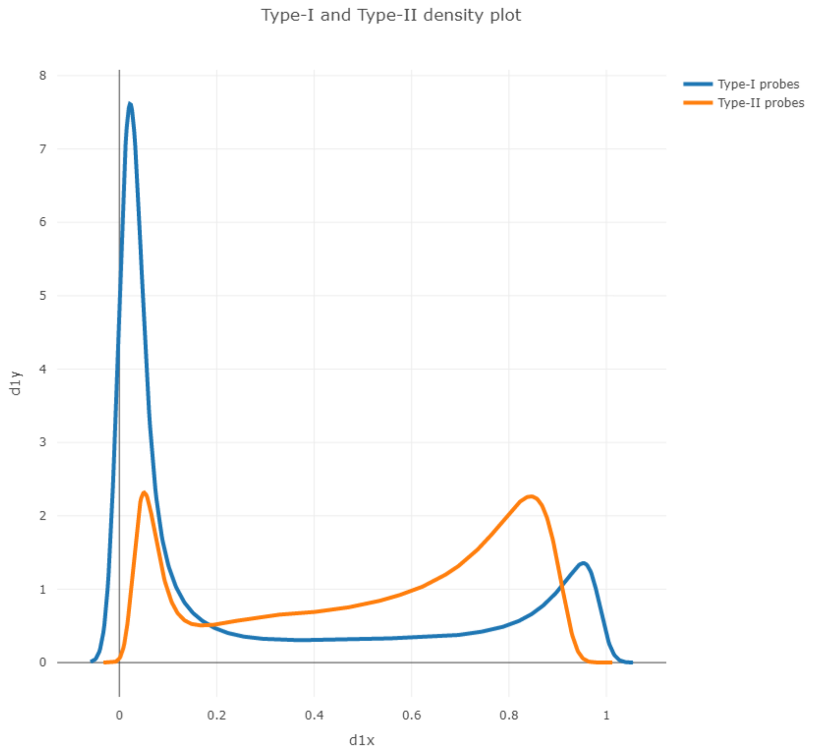

Figure S2 – Post-Normalization Probe Type Density Plot

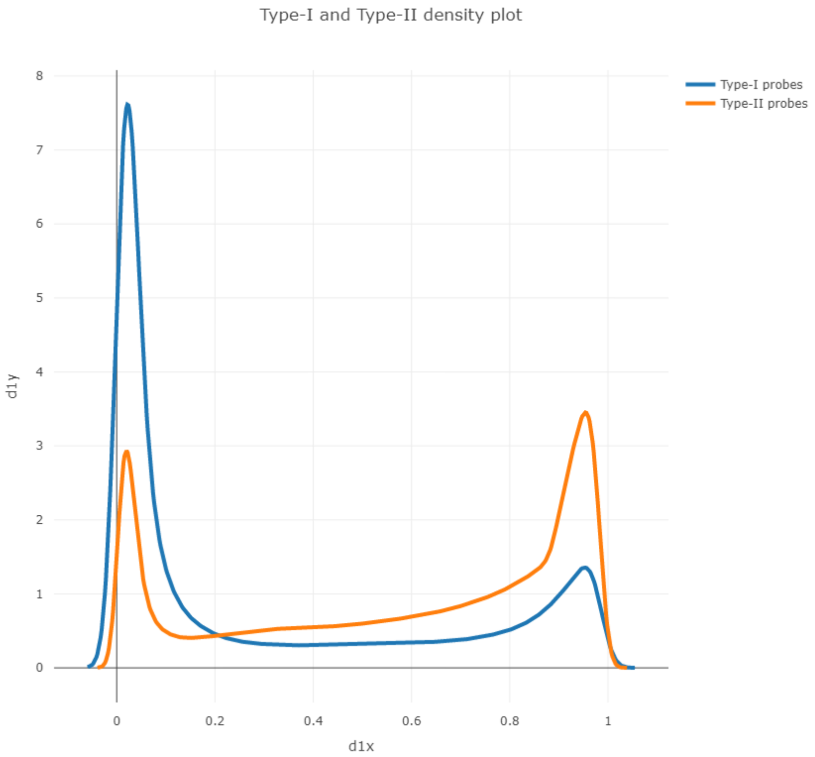

Figure S3 – Pre-Batch Correction Singular Value Decomposition Plot

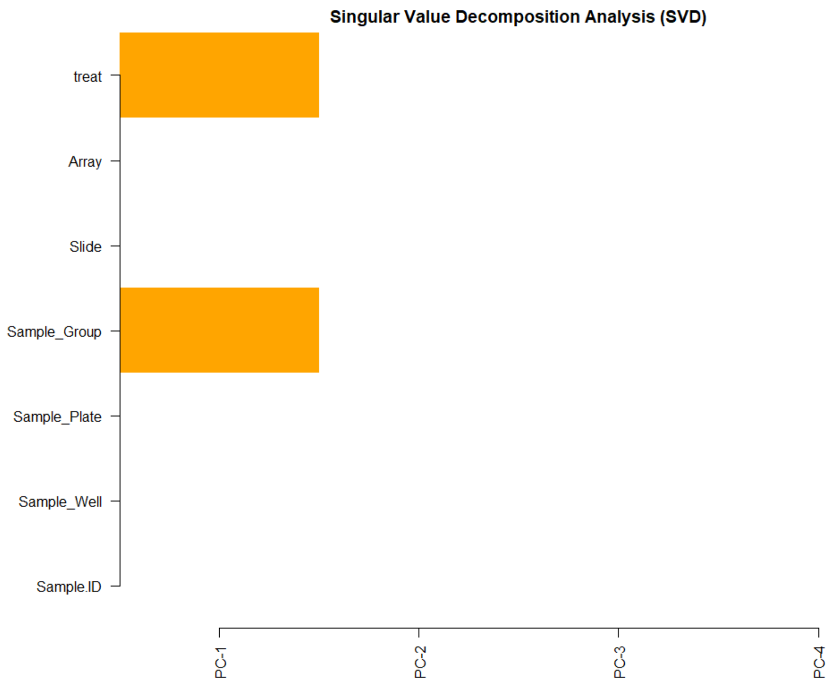

Figure S4 – Post-Batch Correction Singular Value Decomposition Plot

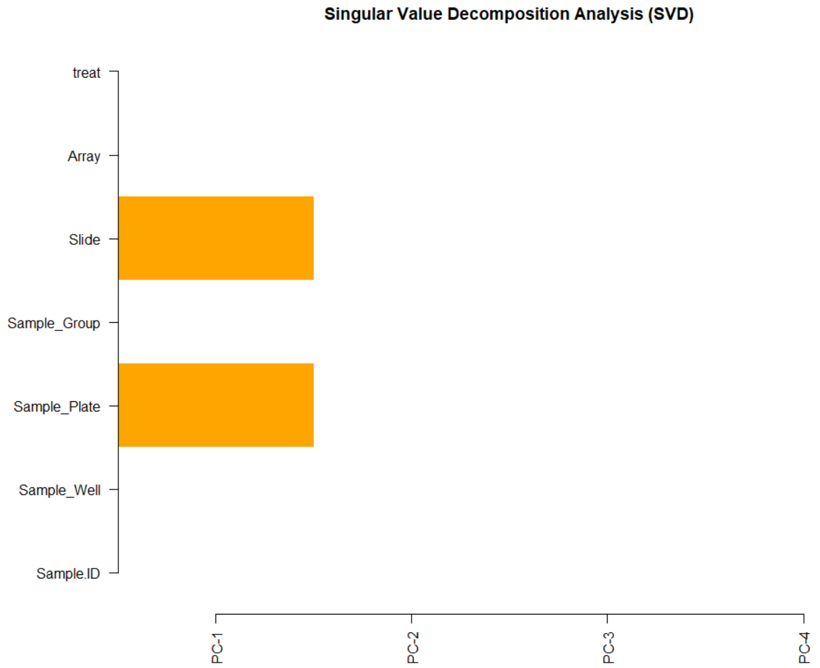

Figure S5 – CG Type Proportions for 97 Significant Sites

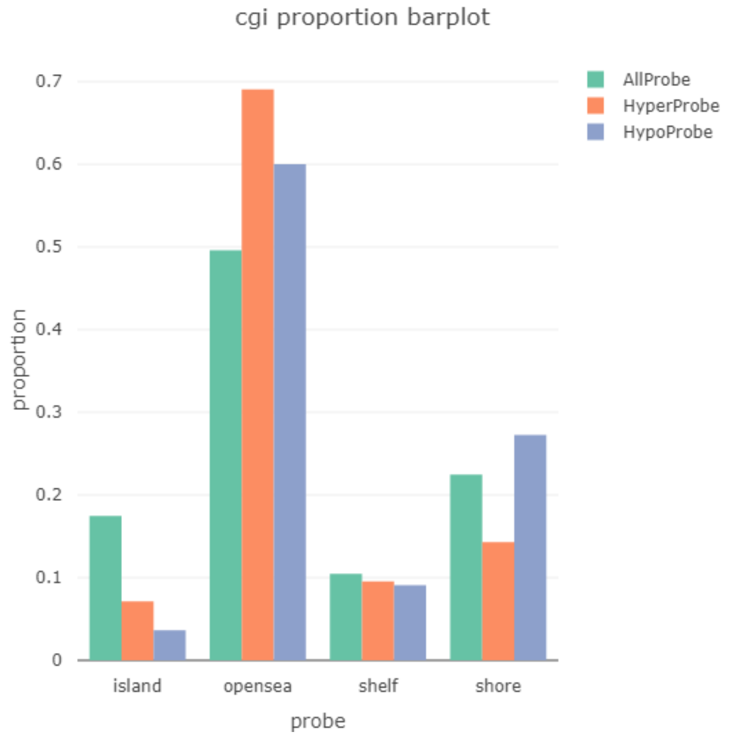

Figure S6 – Gene Feature Proportions for 97 Significant Sites

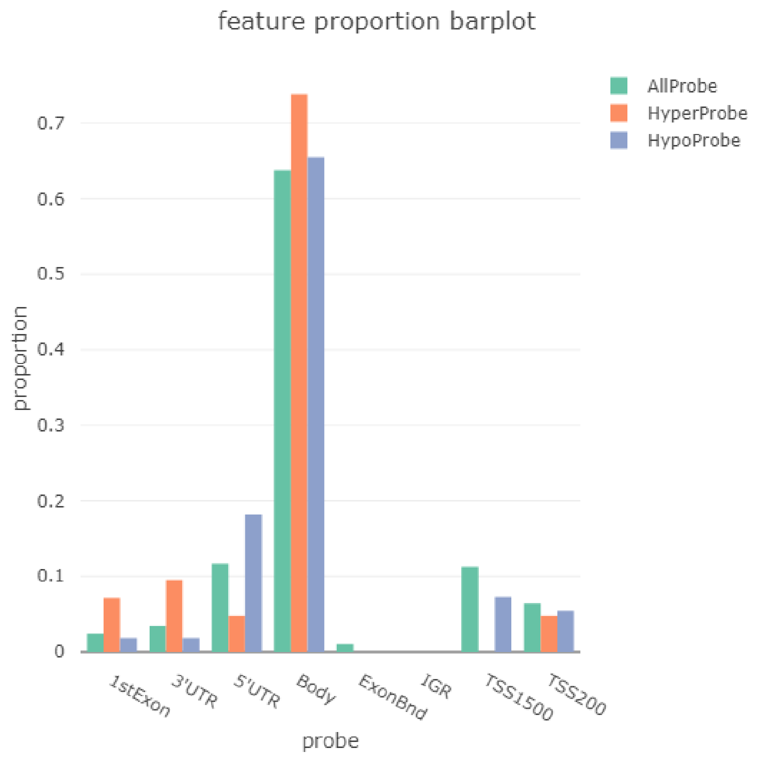

Figure S7 – Gene Methylation Figure for *PRKAR1B*

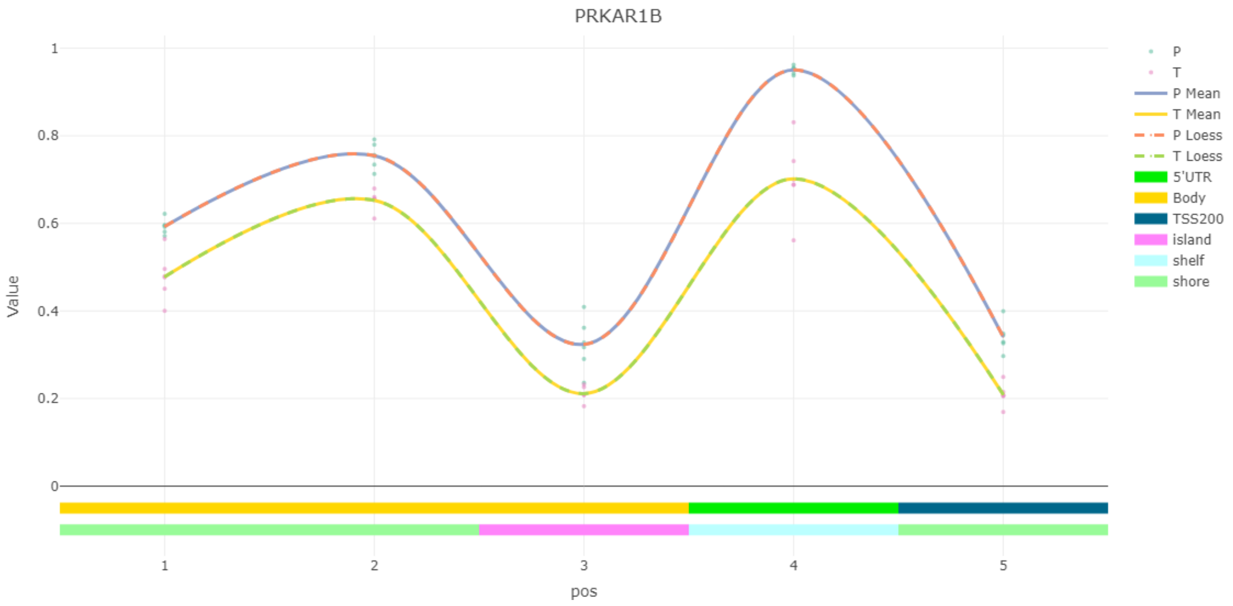

Figure S8 - Gene Methylation Figure for *SREBF1*

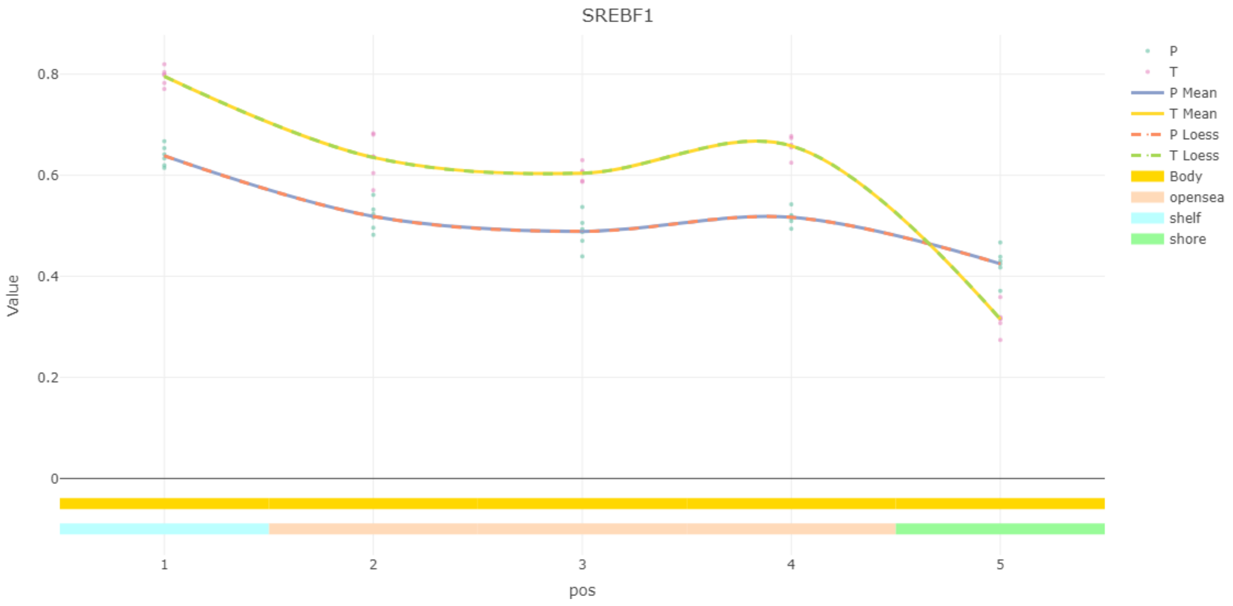

Figure S9 – Gene Methylation Figure for *IRS1*

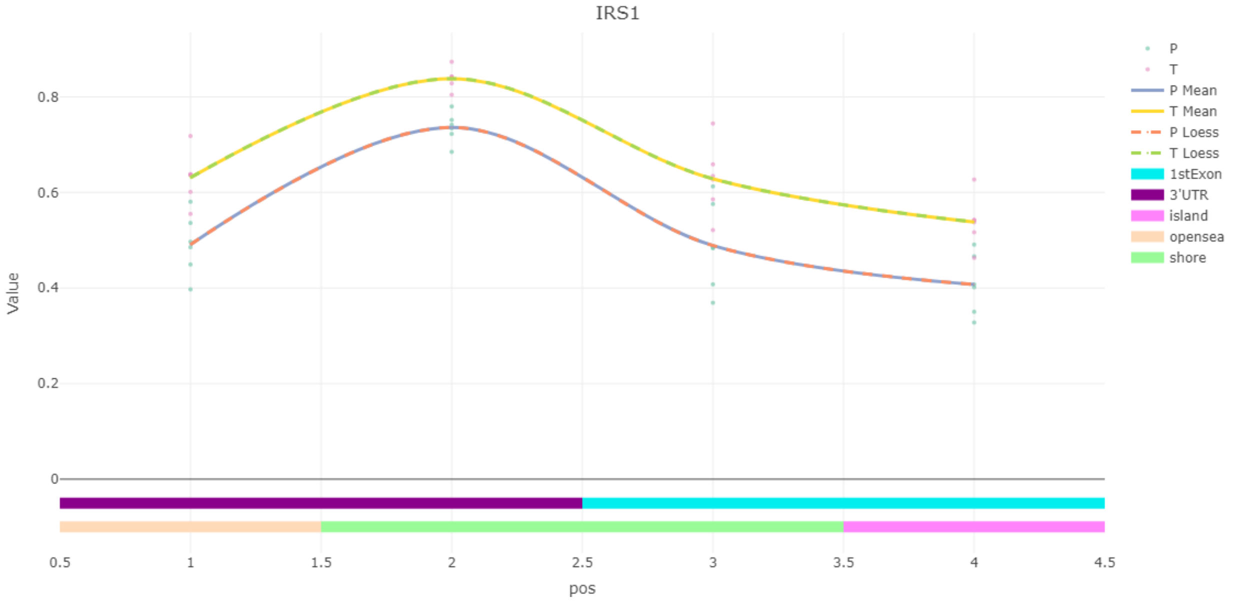

Supplement: Supplementary file 1 [file biomedicines-12-01057-s001.zip › IVGTT_paper_supp_info.pdf]
